# Effect of hBN Particle Size and Content on the Tribological Properties of Polysiloxane-Containing Polyimide Composite Coatings Under Unlubricated Conditions

**DOI:** 10.3390/polym18080948

**Published:** 2026-04-12

**Authors:** Yuelin Fan, Tadashi Shiota

**Affiliations:** 1 Graduate School of Environmental, Life, Natural Science and Technology, Okayama University, Okayama 700-8530, Japan; 2 Faculty of Environmental, Life, Natural Science and Technology, Okayama University, Okayama 700-8530, Japan

**Keywords:** siloxane-containing polyimide, hBN, composite coatings, tribological properties

## Abstract

In this study, polysiloxane-containing polyimide (si-PI) composite coatings containing hexagonal boron nitride (hBN) particles of four different sizes and at different contents were prepared, and their mechanical and tribological properties were investigated. The coatings were deposited on steel substrates via dip coating and cured at 160 °C. Their tribological properties were measured using reciprocating sliding tests under unlubricated conditions against a steel ball. The composite coatings containing nano-hBN with the smallest mean primary particle size of 0.05 μm exhibited the lowest wear. Subsequently, coatings containing 1–15 wt% nano-hBN were prepared to examine the effect of filler content. The results showed that the coatings with low nano-hBN contents (1–2 wt%) had relatively high friction coefficients and significantly reduced wear on both the coating and the counterpart. Cross-sectional scanning electron microscopy (SEM) observations revealed that dispersed small hBN aggregates suppress crack propagation through dispersion strengthening. Coatings with low nano-hBN contents (1–2 wt%) also exhibited sufficient electrical insulation. However, as the hBN content increased further, hBN agglomeration was promoted, weakening the crack-propagation suppression effect and increasing wear. These findings indicate that low-content nano-hBN/si-PI composite coatings are promising electrical erosion-resistant coatings for the outer rings of the bearings used in electric vehicle motors.

## 1. Introduction

In recent years, electric vehicles (EVs) have experienced rapid growth, driven by global carbon neutrality targets [1]. As EVs rely on inverter-driven electric motors that operate under high-frequency switching conditions [2], their bearings in EV motors are exposed to both electrical stress and mechanical loading [3]. This has raised concerns regarding the long-term reliability of the EV motor bearings used in EVs, hybrid electric vehicles (HEVs), and plug-in hybrid electric vehicles (PHEVs) [4].

Reportedly, over 40% of motor failures are related to bearing failures [5,6]. During operation, the shaft voltages generated in electric motors can induce electrical discharges across the bearing lubricant film, leading to morphological damage and lubricant degradation [7,8]. This phenomenon, known as electrical erosion, is a major cause of premature bearing failure [9]. Fluting is a type of electrical erosion characterized by shallow washboard-like grooves on the raceway. It is caused by repetitive discharges associated with motors driven by variable-frequency drives (VFDs), and can lead to premature bearing failure [10]. Creep, a common bearing failure mode, refers to the relative sliding between the bearing’s outer ring and housing or between its inner ring and shaft. This can result in various types of bearing damage, including wear, discoloration, scuffing, and catastrophic failure. Creep is usually caused by insufficient or loose-fitting [11] and is an increasingly important challenge in the design of automotive transmissions [12]. To improve the reliability of bearings in EV motors, it is necessary to develop effective solutions for preventing electrical erosion and creep.

Electrical erosion can be suppressed using two main methods: ceramic- and insulator-coated bearings [13]. Ceramic bearings incorporate ceramic rolling elements or rings and offer excellent insulating properties. However, they are expensive [14]. In insulator-coated bearings, electrical insulation during operation is maintained by coating the bearing outer ring with a high-resistance material, such as alumina. While thermally sprayed alumina coatings exhibit high resistivity and hardness, they inherently possess a porous microstructure, which negatively affects the dielectric breakdown strength [15]. Although increasing coating thickness can enhance dielectric breakdown strength, excessively thick insulating layers can compromise bearing assembly tolerance and dimensional accuracy, negatively affecting fit and performance [16].

Resin coating is a possible alternative to alumina as an insulating coating. In general, resin coatings exhibit limited wear resistance under unlubricated conditions, and the incorporation of inorganic fillers has been widely reported as an effective approach for improving their tribological properties [17,18,19]. Among the various solid lubricants, graphite, hexagonal boron nitride (hBN), and molybdenum disulfide (MoS_2_) are often used because of their intrinsically layered structures and low shear strengths [17]. Compared to graphite (electrically conductive [20]) and MoS_2_ (prone to oxidation at elevated temperatures [21]), hBN provides solid lubrication while maintaining excellent electrical insulation, thermal conductivity, and oxidation resistance [22,23]. These properties make hBN a suitable filler for electrical erosion-resistant resin coatings applied to steel-bearing outer rings. Previous studies on hBN/resin composite coatings have mainly focused on reducing both friction and wear by adding hBN or hybrid fillers [23,24,25]. However, for electrical erosion-resistant coatings on steel bearing outer rings, the coatings must exhibit relatively high friction to prevent creep, while simultaneously maintaining low wear of both the coatings and the bearing housing to ensure durability. This requirement contrasts with those of previous studies, which reported reduced friction.

This study focused on composite coatings that combined a polyimide matrix with a relatively high friction coefficient and hBN particles. This was expected to suppress the wear on both the coating and mating materials while maintaining high friction. Polysiloxane-containing polyimide (si-PI) coatings with insulating and heat-resistant properties have been primarily developed for electronic and aerospace applications. These coatings can be cured at temperatures below 200 °C, equivalent to the tempering temperature of bearing steel. Previous studies have shown that si-PI coatings exhibit low friction and wear under water-lubricated conditions [26]. However, the frictional properties under unlubricated conditions and the effects of hBN addition have not yet been studied. Therefore, in this study, hBN/si-PI composite coatings using hBN particles of various sizes were fabricated, and their friction and wear properties were evaluated under unlubricated conditions. Based on the results, nano-hBN with an average primary particle diameter of 0.05 μm was selected as the filler, and the effect of nano-hBN content on friction and wear behavior was further examined.

## 2. Experimental Method

### 2.1. Preparation of hBN/si-PI Composite Coatings

Commercially available si-PI precursor solutions (SIL-1400, Starfire System Inc., New York, NY, USA) and four types of hexagonal boron nitride (hBN) powders (AP-10S, AP-20S, AP-100S, and AP-170S, MARUKA Co., Ltd., Ena, Japan) were used to prepare hBN/si-PI composite coatings. The mean primary particle diameters of the hBN powders provided by the manufacturer are summarized in Table 1. However, as seen in the scanning electron microscopy (SEM) images, BN3.0 and BN0.7 appear to be consistent with the nominal particle sizes, whereas BN0.1 and BN0.05 tend to exhibit some agglomeration. The median and mean values for each powder were measured using a particle size distribution analyzer (LA960S2, Horiba Ltd., Kyoto, Japan) and were found to be 3.9 and 4.3 μm for AP-10S, 2.6 and 5.0 μm for AP-20S, 5.3 and 9.1 μm for AP-100S, and 2.1 and 6.7 μm for AP-170S, respectively. The composite coatings fabricated using hBN as a filler were named BN3.0, BN0.7, BN0.1, and BN0.05, respectively (Table 1). For comparison, a si-PI coating without hBN fillers was also fabricated and named N.

**Figure 1:**
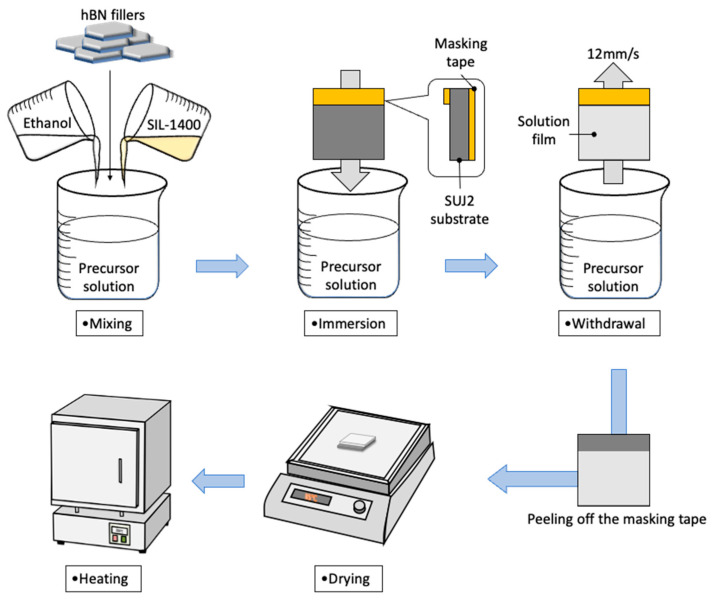
Dip coating process in this study.

The si-PI precursor solution was diluted with 99.5 wt% ethanol at a mass ratio of 3:2 to adjust the viscosity for dip-coating. Subsequently, each hBN powder was added to the diluted PI solution. The mixture was stirred for 15 min, then ultrasonicated for 20 min to achieve homogeneous dispersion of the hBN fillers within the si-PI matrix. The coating was performed using a dip-coating method, as shown in Figure 1. A bearing steel (SUJ2, Japanese Industrial Standards [JIS]) plate measuring 15 mm × 15 mm × 1.6 mm, with a mirror-polished surface and a surface roughness of approximately 0.15 μmRa, was used as a coating substrate. In addition, a Si (001) substrate measuring 15 mm × 15 mm × 0.635 mm with a surface roughness of 0.001 μmRa was used to facilitate detailed observation of the coating cross-sections before and after the sliding test. Before coating, the substrates were cleaned using an ethanol-based solvent mixture in an ultrasonic cleaner to remove surface contaminants. A portion of the substrate was covered with masking tape before dip coating to determine the coating thickness by measuring the height difference between the coating and the substrate. After cleaning, the substrate was immersed in the coating solution for 60 s, then withdrawn at a constant speed of 12 mm/s. The coated substrate was placed horizontally and dried at 85 °C in air for 2 h on a heating plate. The masking tape was peeled off after drying. Finally, the coated substrate was placed in an electronic furnace and cured at 160 °C, a typical tempering temperature for bearing steels. The heating rate was set to be 1 °C/min, and the curing temperature was maintained for 2 h. After completion of the curing process, the samples were kept in the electronic furnace for approximately 12 h to allow natural cooling to room temperature (20–25 °C).

### 2.2. Measurement of the hBN/si-PI Composite Coating Properties

A surface profiler (FORMTRACER FTA-S4S3000; Mitutoyo, Kawasaki, Japan) was used to determine the coating thickness. The surface roughnesses of the coatings were measured simultaneously. The hardness and Young’s modulus were measured using a Berkovich diamond nanoindenter (ENT-5, ELIONIX Inc., Hachioji, Japan). The indentation depth was set to 1 μm, which was less than 10% of the coating thickness. The indenter was pushed into a depth of 1 μm at a constant loading rate of 0.15 mN/s, held for 1000 ms, and then retracted at the same loading rate. Hardness was calculated using the Oliver-Pharr method. Measurements were performed at 25 points at 50 μm intervals, and the average value and standard deviation were recorded. The surface and internal structures of the coatings were observed using laser microscopy and field-emission scanning electron microscopy (FE-SEM; JSM-IT800, JEOL Ltd., Akishima, Japan).

**Figure 2:**
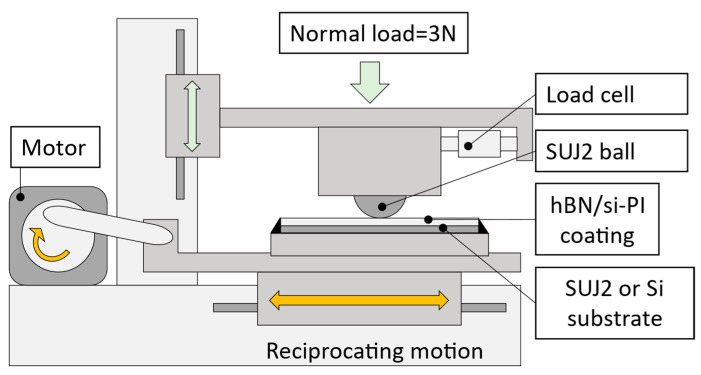
A schematic drawing of a home-built tribometer used in this study.

To study the tribological properties of these coatings under unlubricated conditions, reciprocating sliding tests were conducted at room temperature (20–25 °C) in air. A schematic of the sliding testing machine is shown in Figure 2. The normal load, average sliding speed, and sliding distance (sliding time) were set as 3 N, 20 mm/s, and 6 m (5 min), respectively. A SUJ2 ball (Grade 28) with a diameter of 10 mm and surface roughness of 0.05 μmRa was used as a mating material. In the sliding test of the coating on the SUJ2 substrate, the sliding direction was set perpendicular to the polishing marks on the substrate. After the sliding tests, the friction surfaces were observed using FE-SEM to determine the wear type. The cross-sectional profile, including the wear scar, was measured using a surface profiler to estimate the specific wear rates of the coatings. The specific wear rates of the coatings were calculated using Equation (1),

(1) $$W=\frac{V}{F\times L}=\frac{S\times d\times 1000}{F\times T\times f\times d\times 2}$$

where W is the specific wear rate ($mm^{3}N^{-1}m^{-1}$), V is the wear volume ($mm^{3}$), F is the normal load (N), L is the sliding distance (m), T is the sliding time (s), d is the sliding stroke (mm), S is the average wear scar cross-sectional area ($mm^{2}$), f is the sliding frequency (Hz).

## 3. Results and Discussion

### 3.1. Selection of hBN Fillers

The tribological properties of the N, BN3.0, BN0.7, BN0.1, and BN0.05 coatings under unlubricated conditions were compared to select the best candidate filler that would improve their properties. The concentration of all hBN fillers in the coating solution was maintained at approximately 13 wt%. The average coating thickness was approximately 22–33 μm and showed particle-size dependence as shown in Figure S1 of the supplemental materials. At the same filler concentration, smaller particles generally led to a more pronounced increase in solution viscosity than larger particles [27]. In the dip coating method, a thicker coating is typically produced with a higher solution viscosity [28]. The moderate particle size (0.7 μm) likely provided an optimal balance between surface area and dispersion stability, thereby increasing the suspension viscosity and leading to a thicker deposited layer. However, when the particle size was further reduced, the coating thickness decreased. This deviation from the expected trend is attributed to the agglomeration of the nano-hBN particles, as observed in the SEM images in Table 1. Agglomeration reduces the effective dispersion of particles in the solution and suppresses the viscosity-enhancing effect of well-dispersed nanosized fillers, thereby leading to a smaller coating thickness.

**Figure 3:**
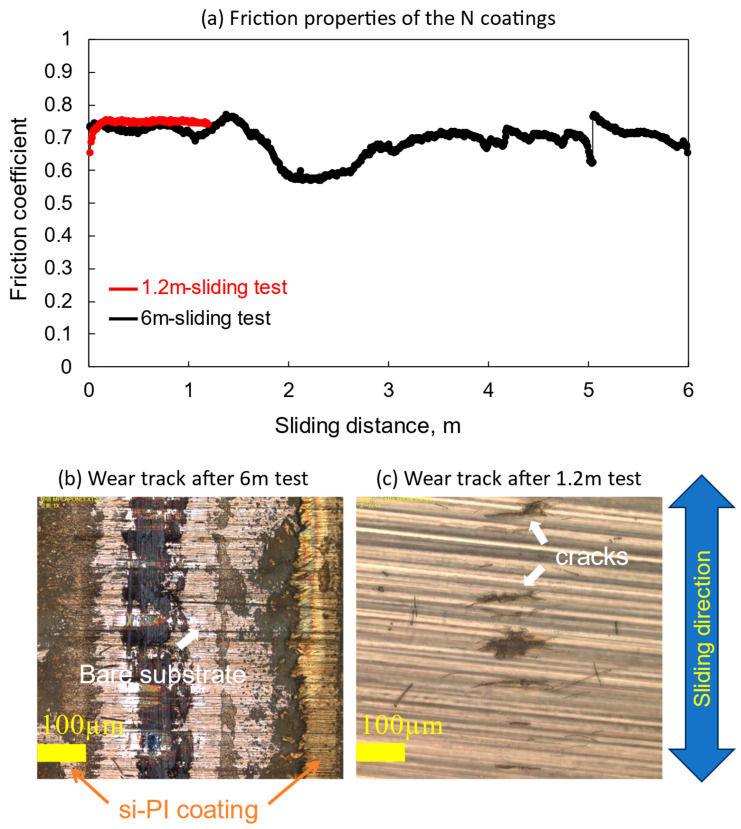
Friction properties and surface observation images of the N coatings after the sliding test.

Figure 3 shows the friction properties of the N coatings based on the wear track observation results. The friction coefficient fluctuated during the 6 m sliding test, as shown by the black line in Figure 3a, and the coating was partially peeled and severely damaged, as shown in Figure 3b. To study the damage process, an additional sliding test was conducted with a shorter sliding distance of 1.2 m. The friction properties and wear track observation results are indicated by the red lines in Figure 3a and Figure 3c, respectively. The friction coefficient remained stable throughout the tests. However, several transverse cracks with a length of approximately 100 μm can be seen on the friction surface. These cracks propagated and coalesced, leading to early partial peeling and severe damage to the coating.

**Figure 4:**
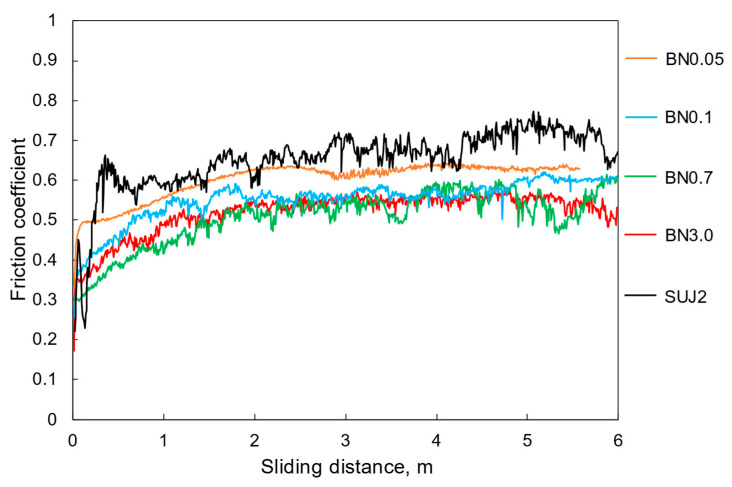
Friction properties of the hBN/si-PI coatings with different hBN sizes as a function of the sliding distance.

**Figure 5:**
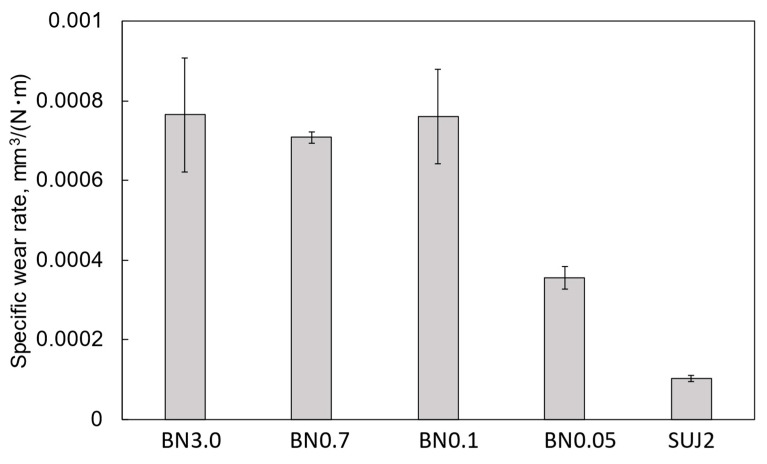
Specific wear rates of the hBN/si-PI composite coatings with different hBN sizes and the SUJ2 substrate under unlubricated conditions.

Figure 4 shows the friction properties of the composite coatings. For comparison, the results for the self-mated SUJ2 friction are also included. The SUJ2 substrate exhibited the highest average friction coefficient (approximately 0.68). The BN0.05 coating exhibited the second-highest average friction coefficient of approximately 0.60 and the most stable friction characteristics. This stable friction was likely due to the reduction in the surface roughness of the friction surface due to the introduction of nano-hBN particles. Figure 5 shows the specific wear rates of the coatings calculated from the cross-sectional curves in Figure S2. Despite having the thinnest average coating thickness among the composite coatings, the BN0.05 coating exhibited the least wear, indicating that the coating thickness is not the primary factor determining the wear behavior in this study. The superior wear resistance of the BN0.05 coating is likely related to the reduction in surface roughness and the crack-propagation suppression effect of the fine hBN particles, which will be discussed later.

For the application of electrical erosion-resistant coatings deposited on the bearing outer rings, high friction is essential to suppress bearing creep, and low wear is essential to preserve coating integrity during long-term service. From this perspective, the BN0.05 coating is the best candidate for such applications because it showed relatively high friction and the lowest specific wear rate among the composite coatings. This result is consistent with previous studies reporting that smaller hBN particles tend to enhance the wear resistance because of their more uniform dispersion in the polymer matrix [29,30].

### 3.2. Preparation and Characterization of the Nano-hBN/si-PI Composite Coatings

Nano-hBN/si-PI composite coatings (BN0.05) with different filler contents were prepared to study the effect of adding nano-hBN on their mechanical and tribological properties. Five sets of BN0.05 coating solutions with nano-hBN concentrations of 1, 2, 5, 10, and 15 wt% were prepared. These coating solutions were used to fabricate composite coatings on Si substrates, which are referred to as BN0.05_1, BN0.05_2, BN0.05_5, BN0.05_10, and BN0.05_15 (Table 2).

**Figure 6:**
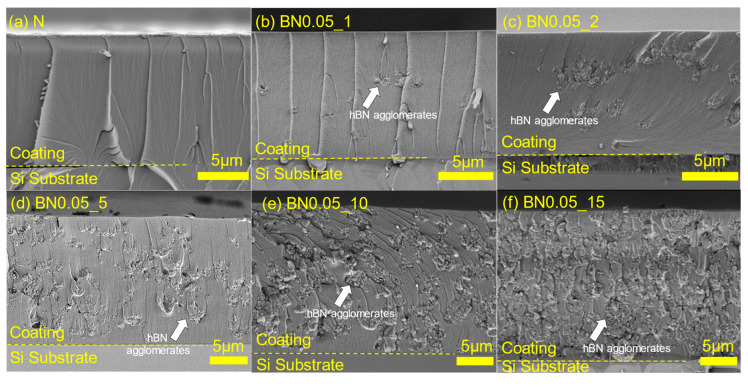
Cross-sectional images of the nano-hBN/si-PI composite coatings with different hBN contents. The white arrows indicate examples of hBN aggregates.

**Figure 7:**
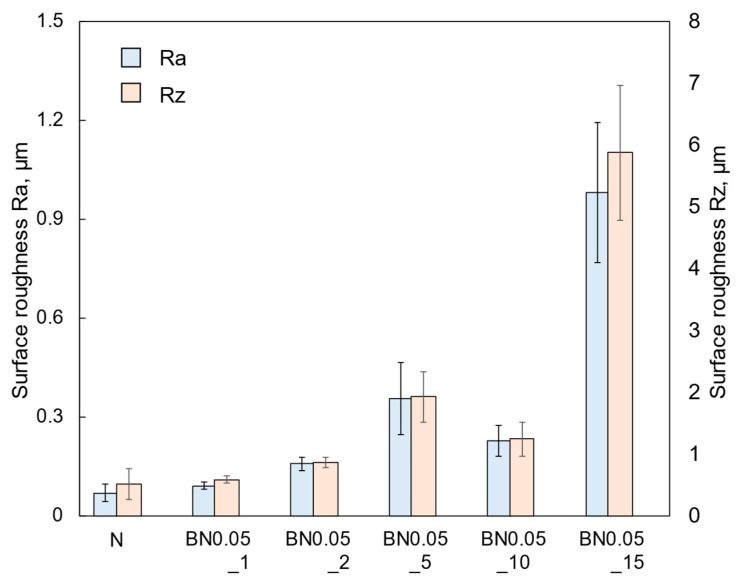
Surface roughnesses Ra and Rz of the nano-hBN/si-PI composite coatings with different hBN contents.

For comparison, a si-PI coating without hBN addition (N coating) was also fabricated on a Si substrate. Figure 6 shows cross-sectional SEM images of the coatings. The observation samples were prepared by scribing and breaking; however, the cross-sections were not polished. Several cracks along the thickness direction can be observed in the N coating. These cracks are introduced during preparation of the observation samples, indicating that the si-PI matrix is relatively brittle. Similar cracks are also visible in the BN0.05_1 coating. Furthermore, when the hBN content is low (e.g., BN0.05_1 and BN0.05_2 coatings), small nano-hBN agglomerations are sparsely distributed, but as the hBN content increases, both the density and size of the agglomerates increase (see Figure S3). This agglomeration behavior is likely owing to the high surface energy of the nanosized particles [31], which prevented sufficient de-agglomeration by the ultrasonic dispersion treatment. The coating thickness increased with the hBN content from 12 to 25 μm (see Figure S4). This trend can be attributed to the increased solution viscosity caused by the higher hBN content, which leads to a greater amount of material being deposited on the substrate during dip coating [32]. The arithmetic average roughness (Ra) and maximum height roughness (Rz) of the coatings are shown in Figure 7. Both Ra and Rz increased with the hBN content. This is caused by the increase in the density and size of hBN agglomerates beneath the coating surfaces, as can be seen in Figure 6.

### 3.3. Mechanical and Tribological Properties of the Nano-hBN/si-PI Composite Coatings

**Figure 8:**
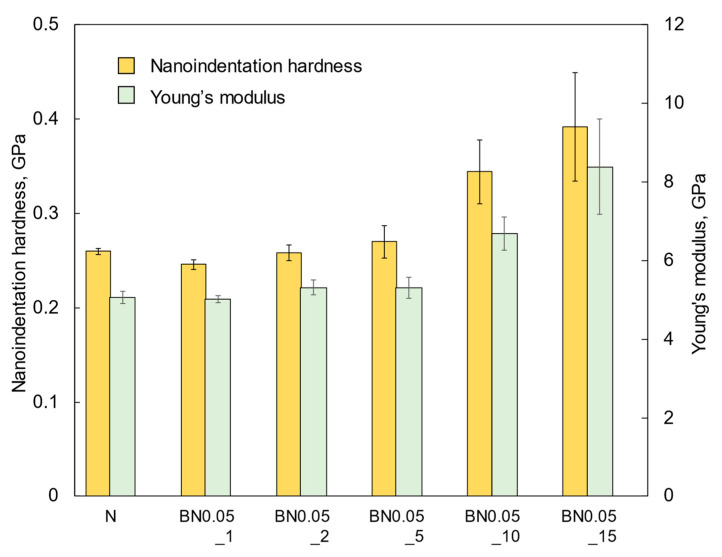
Nanoindentation hardness and Young’s modulus of the nano-hBN/si-PI composite coatings with different hBN contents.

The nanoindentation hardness and Young’s modulus values of the BN0.05 composite coatings are shown in Figure 8. The nanoindentation hardness of the N coating was consistent with a previous report [26] and with that of conventional polyimide (Vespel SP-1, DuPont de Nemours, Inc., Wilmington, DE, USA). The hardness of BN0.05 coatings increased with increasing hBN content. This is attributed to the increase in the density and size of the hBN agglomerates embedded within the si-PI matrix, which had a higher hardness.

**Figure 9:**
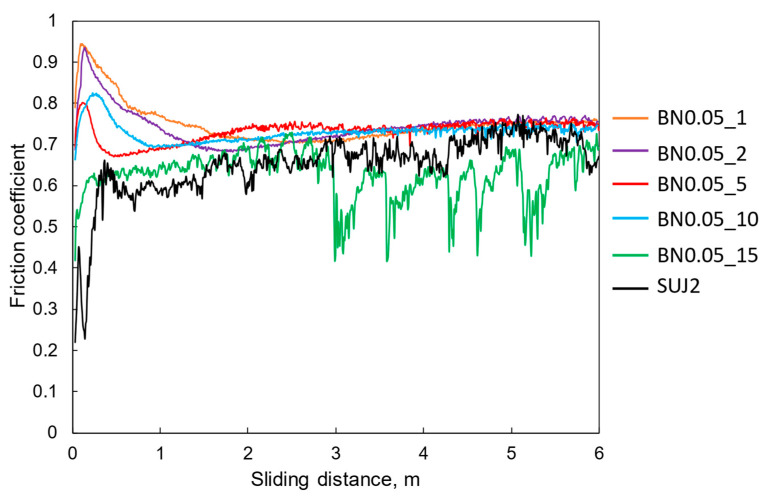
Friction coefficient of the nano-hBN/si-PI composite coatings with different hBN contents and the SUJ2 substrate as a function of the sliding distance under unlubricated conditions.

Figure 9 shows the friction coefficient as a function of the sliding distance. For reference, the friction properties of the self-mated SUJ2 contacts are also included. Except for the BN0.05_15 coating, the friction coefficient increased and then decreased within the first 1 m of the sliding distance, and then increased slowly to reach a steady state. For the BN0.05_15 coating, the friction coefficient increased gradually in the early stages and fluctuated significantly in the later stages. This is attributed to the formation and detachment of the transfer film, as described below. As shown in Figure 9, the BN0.05 composite coatings with hBN contents of 10 wt% or less exhibited higher average friction coefficients than those of the self-mated SUJ2 contact.

**Figure 10:**
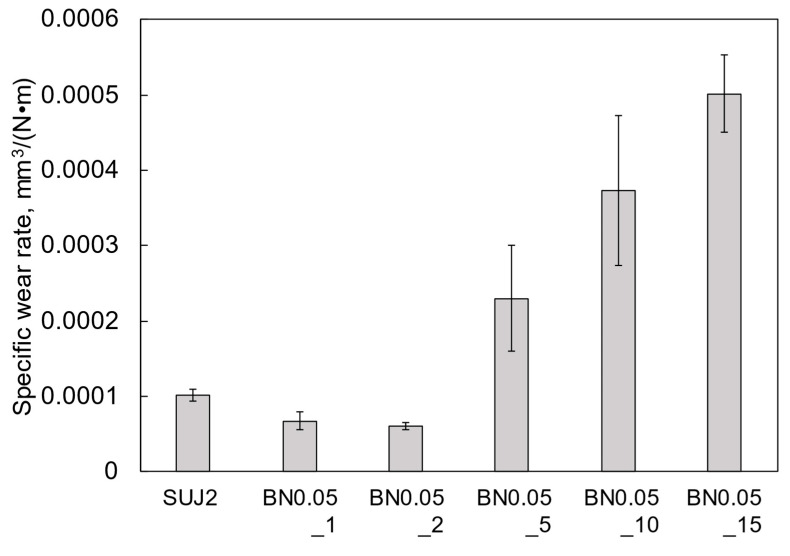
Specific wear rates of the nano-hBN/si-PI composite coatings with different hBN contents and the SUJ2 substrate under unlubricated conditions.

**Figure 11:**
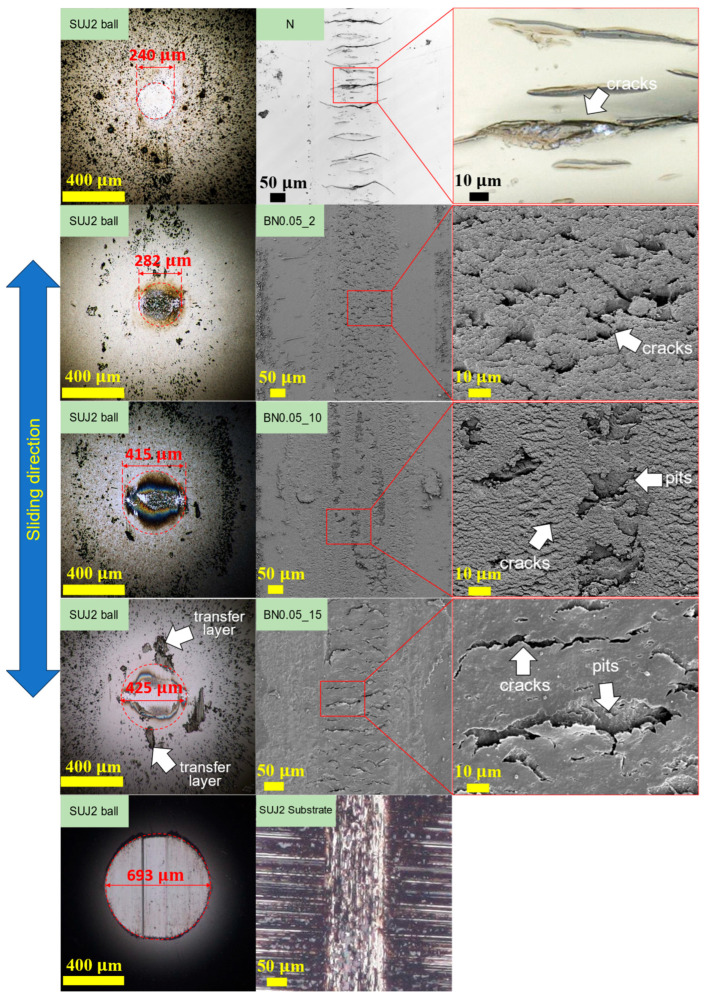
Worn surface morphologies of the nano-hBN/si-PI composite coatings with different hBN contents, the SUJ2 substrate, and the corresponding mating SUJ2 balls under unlubricated conditions.

As shown in Figure S5, the wear depths of the BN0.05_1 and BN0.05_2 coatings were similar, both approximately 2 μm. The wear depth increased with the hBN content, and the BN0.05_15 coating exhibited the largest wear depth, approximately 10 μm. Consequently, the specific wear rate shown in Figure 10 was the highest for the BN0.05_15 coating, whereas the BN0.05_2 coating showed the lowest, which was lower than that of the SUJ2 substrate. Figure 11 shows the wear tracks of the BN0.05_2, BN0.05_10, and BN0.05_15 coatings, N coating, and SUJ2 substrate, along with the corresponding mating ball surfaces. Many long transverse cracks with lengths over 100 μm appeared on the friction surface of the N coating, which is similar to Figure 3c. These large cracks caused premature peeling and extensive damage to the N coating, as described above. By contrast, numerous cracks with lengths of less than approximately 20 μm can be observed on the friction surfaces of the BN0.05_2 and BN0.05_10 coatings. These cracks were smaller than those on the N coating, indicating that the incorporation of nano-hBN fillers suppressed crack propagation by friction. However, the friction surface of the BN0.05_15 coating has many cracks longer than 50 μm, suggesting that when the hBN content becomes high, the crack propagation suppression effect is weakened. In addition, several pits are observed on the friction surfaces of the BN0.05_10 and BN0.05_15 coatings. The diameters of these pits increased with the hBN content and were comparable to those of the hBN agglomerates in these coatings, suggesting that the pits were likely formed by the detachment of the hBN agglomerates due to friction. Thus, the density and size of the hBN agglomerates in the coating increased with increasing hBN content, resulting in a reduction in the crack-propagation suppression effect and an increase in the detachment of the hBN agglomerates by friction. Therefore, the specific wear rate increases with increasing hBN content.

Wear scars were clearly observed on the surfaces of all mating SUJ2 balls, as shown in Figure 11. The diameter of the wear scar on the mating ball increased with the hBN content in the coating. The composite coatings with higher hBN content released more hBN agglomerates into the frictional interface, leading to increased wear of the mating ball. Moreover, a noticeable transfer layer was observed only on the mating ball against the BN0.05_15 coating. Because of the detached hBN agglomerates and the unstable transfer layer, the friction coefficient of the BN0.05_15 coating was low and unstable, as shown in Figure 9. The diameter of the wear scar on the mating ball against the SUJ2 substrate is larger, which is more than twice as large as that against the BN0.05_2 coating. Thus, the BN0.05_2 coating also reduced the wear on the mating material.

Coatings deposited on the outer rings of the bearing require a high friction coefficient, high wear resistance, and low aggressiveness of the bearing housing. However, previous studies on various hBN/resin composite coatings have primarily focused on reducing both friction and wear through the addition of hBN [22,33,34]. By contrast, this study showed that a nano-hBN/si-PI composite coating with a small amount (2 wt%) of nano-hBN (BN0.05_2 coating) exhibited a higher friction coefficient, lower wear, and lower aggressiveness toward the mating material. Moreover, the insulating property of the BN0.05_2 coating was evaluated using the measurement system shown in Figure S6. Even when DC 400 V was applied in the thickness direction of the coating for 60 s, the measured current remained below the detection limit of the ammeter (FLUKE 287 multimeter, Fluke co., Everett, WA, USA), which is 0.01 μA. This result shows that the resistance of the BN0.05_2 coating is 40 GΩ or higher, indicating sufficient insulating properties. Therefore, the BN0.05_2 coating is a promising electrical erosion-resistant coating for deposition on the bearing outer rings.

### 3.4. Wear Mechanism of the Nano-hBN/si-PI Composite Coatings

**Figure 12:**
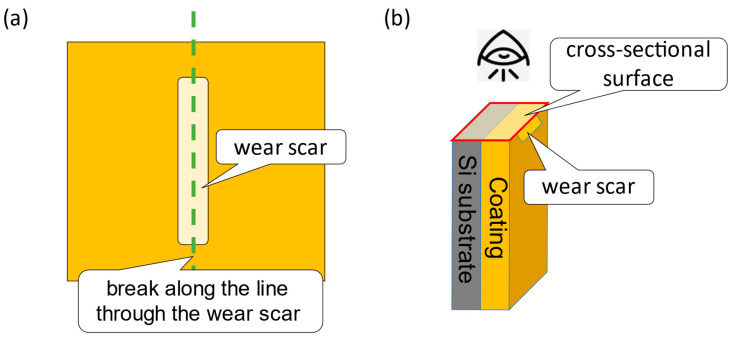
Schematic illustration of preparation for the cross-sectional observation sample along the direction of the wear scar; (**a**) scribed line (green line) and (**b**) observation direction.

**Figure 13:**
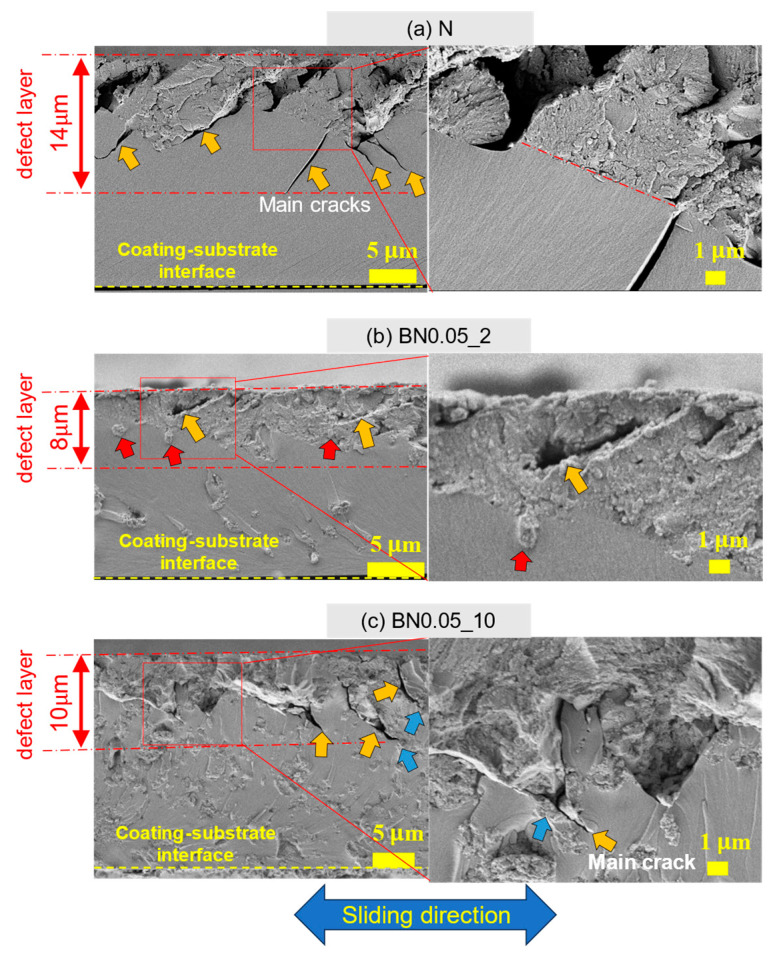
Cross-sectional images including the wear scars of the nano-hBN/si-PI coatings under unlubricated conditions. Orange, red and blue arrows indicate main cracks, small hBN agglomerates beneath or along the crack paths, and larger hBN agglomerates penetrated by main cracks.

**Figure 14:**
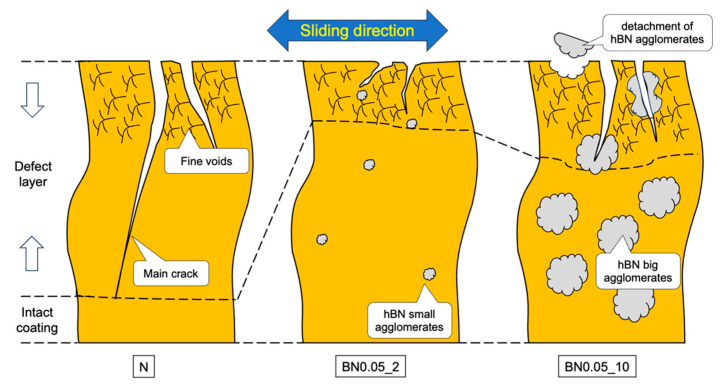
A wear mechanism model for the nano-hBN/si-PI coatings with different hBN contents.

According to the tribological properties of the composite coatings, the BN0.05_2 coating exhibited the best wear resistance and a relatively high friction coefficient. To better understand the wear mechanism, the N, BN0.05_2, and BN0.05_10 coating samples were scribed and broken along the direction of the wear track, as shown in Figure 12. The cross-sections, including their friction surfaces, were observed using FE-SEM. The results are summarized in Figure 13. The subsurface beneath the friction surface of the coating contained main cracks (indicated by the orange arrows) and numerous fine voids. This region is referred to as the “defect layer.” However, the structure beneath the defect layer remains intact. The N coating has a thicker defect layer containing a greater number of main cracks with longer crack lengths than the BN0.05_2 and BN0.05_10 coatings. By contrast, the propagation of the main cracks in the BN0.05_2 and BN0.05_10 coatings was significantly influenced by the nano-hBN particles and their agglomerates. In the BN0.05_2 coating, the main cracks did not show a clear tendency to extend toward the interior or the interface between the coating and substrate. Instead, small hBN agglomerates (indicated by red arrows) are observed beneath or along the crack paths, suggesting that crack propagation is inhibited or deflected by the dispersed nano-hBN particles. However, in the BN0.05_10 coating, some main cracks penetrated the larger hBN agglomerates (indicated by blue arrows), indicating that larger agglomerates became more prone to fracture, weakening their crack-arresting effect. Based on these observations, wear mechanisms of the nano-hBN/si-PI composite coatings were proposed, as shown in Figure 14. In the N coating, the main cracks can readily propagate into the interior of the coating and reach the coating-substrate interface under friction owing to the brittle nature of the si-PI material, leading to the formation of large cracks and premature, extensive damage to the coating, as shown in Figure 3b,c and Figure 11. In nano-hBN/si-PI composite coatings with a low hBN content, main cracks tend to deflect toward the hBN particles and their small agglomerates [35]. The propagating cracks are arrested or deflected by these particles and small agglomerates. This crack-arrest and deflection effect induced by dispersed hBN particles and small agglomerates is commonly referred to as dispersion toughening [35]. However, as the hBN content and the size of the hBN agglomerates increased, the larger agglomerates became more susceptible to fracture by crack propagation, weakening the dispersion-toughening effect and leading to partial damage to the coating. Moreover, because larger agglomerates are easily detached from the surface under friction, the wear of the composite coatings with higher hBN content becomes more severe.

## 4. Conclusions

This study revealed that the addition of hBN fillers significantly affected the tribological properties of si-PI coatings under unlubricated conditions. The effect of four types of hBN fillers on tribological performance was investigated, and nano-hBN fillers with an average primary particle size of 0.05 μm exhibited the best wear resistance. Nano-hBN/si-PI composite coatings with low hBN content (1–2 wt%) exhibited a relatively high friction coefficient, comparable to that of bearing steel. However, the specific wear rate was lower than that of the bearing steel substrate, and the wear on the mating-bearing steel balls was reduced. Furthermore, sufficient electrical insulation was also confirmed. Therefore, the nano-hBN/si-PI composite coating developed in this study is a promising candidate as an electrical erosion-resistant coating for steel-bearing outer rings where a high friction coefficient, high wear resistance, and electrical insulation are required.

## Figures and Tables

**Table 1:** hBN powders with different sizes used in this study.

Mean Primary Particle Diameters	3.0 μm	0.7 μm	0.1 μm	0.05 μm
SEM images	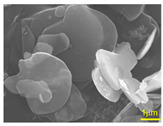	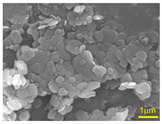	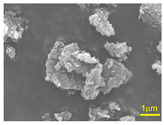	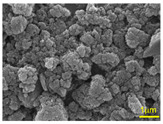
Coating name	BN3.0	BN0.7	BN0.1	BN0.05

**Table 2:** Coating names of the nano-hBN/si-PI composite coatings with different hBN contents.

Nano-hBN Content, wt%	0	1	2	5	10	15
Coating name	N	BN0.05_1	BN0.05_2	BN0.05_5	BN0.05_10	BN0.05_15

## Data Availability

The original contributions of this study are included in this article. Further inquiries can be directed to the corresponding authors.

## Supplementary Materials

The following supporting information can be downloaded at: https://www.mdpi.com/article/10.3390/polym18080948/s1, Figure S1: Average coating thickness of hBN/si-PI composite coatings with different hBN sizes. The error bars indicate the standard deviation; Figure S2: Cross-sectional profiles of the wear scars of the hBN/si-PI composite coatings with different hBN sizes under unlubricated conditions; Figure S3: Example of elemental distribution analysis in a cross-sectional area. This is the case for the BN0.05_5 coating. Since signals for N and B were detected in the area indicated by the white arrows, it can be concluded that these are aggregates of hBN; Figure S4: Average coating thickness of nano-hBN/si-PI composite coatings with different nano-hBN contents. The error bars indicate the standard deviation; Figure S5: Cross-sectional profiles of the wear scars of the nano-hBN/si-PI composite coatings with different hBN contents and the SUJ2 substrate under unlubricated conditions; Figure S6: Measurement setup to evaluate electrical insulation of the hBN/si-PI composite coating.
