# Supplementary material for: Effect of hBN Particle Size and Content on the Tribological Properties of Polysiloxane-Containing Polyimide Composite Coatings Under Unlubricated Conditions
Source: Polymers (Basel). 2026 Apr 12;18(8):948. doi: 10.3390/polym18080948 (PMC13120450; doi:10.3390/polym18080948)
Supplement: Supplementary file 1 [file polymers-18-00948-s001.zip › polymers-4146177-supplementary.pdf]

Supplementary Materials for

Effect of hBN Particle Size and Content on the Tribological Properties of Polysiloxane-Containing Polyimide Composite Coatings Under Unlubricated Conditions

Yuelin Fan [1] and Tadashi Shiota [2,*]

[1] Graduate School of Environmental, Life, Natural Science and Technology, Okayama University, Okayama 700-8530, Japan
[2] Faculty of Environmental, Life, Natural Science and Technology, Okayama University, Okayama 700-8530, Japan
* Correspondence: t-shiota@okayama-u.ac.jp

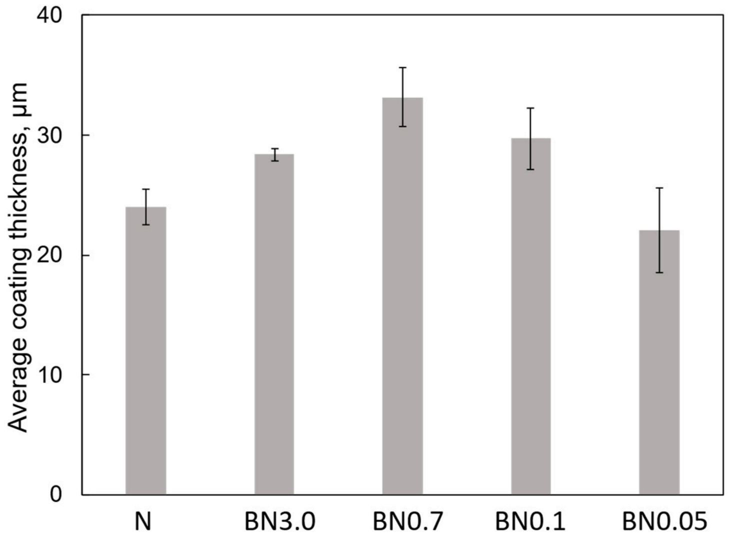

Figure S1. Average coating thickness of hBN/si-PI composite coatings with different hBN sizes. The error bars indicate the standard deviation.

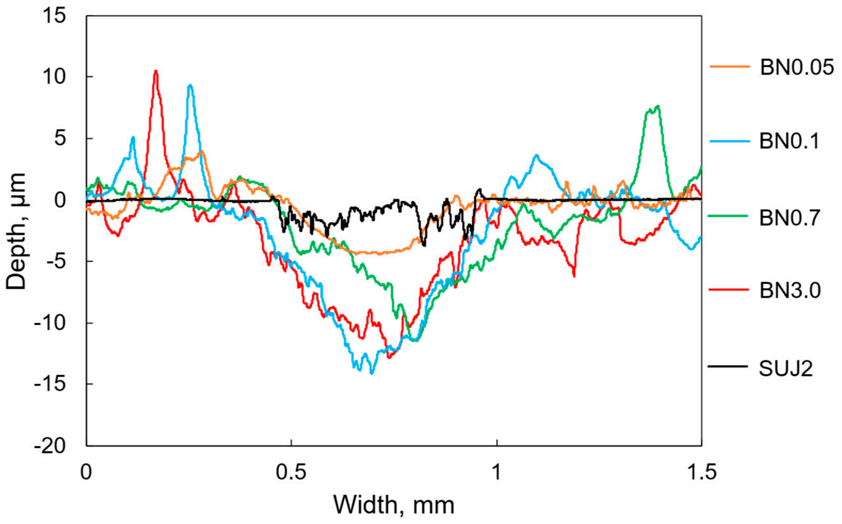

Figure S2. Cross-sectional profiles of the wear scars of the hBN/si-PI composite coatings with different hBN sizes under unlubricated conditions.

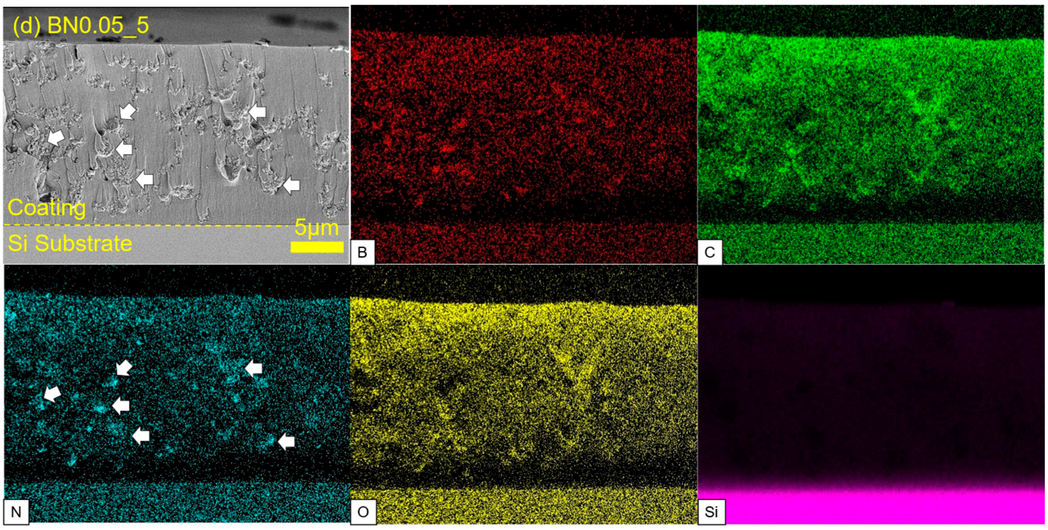

Figure S3. Example of elemental distribution analysis in a cross-sectional area. This is the case for the BN0.05_5 coating. Since signals for N and B were detected in the area indicated by the white arrows, it can be concluded that these are aggregates of hBN.

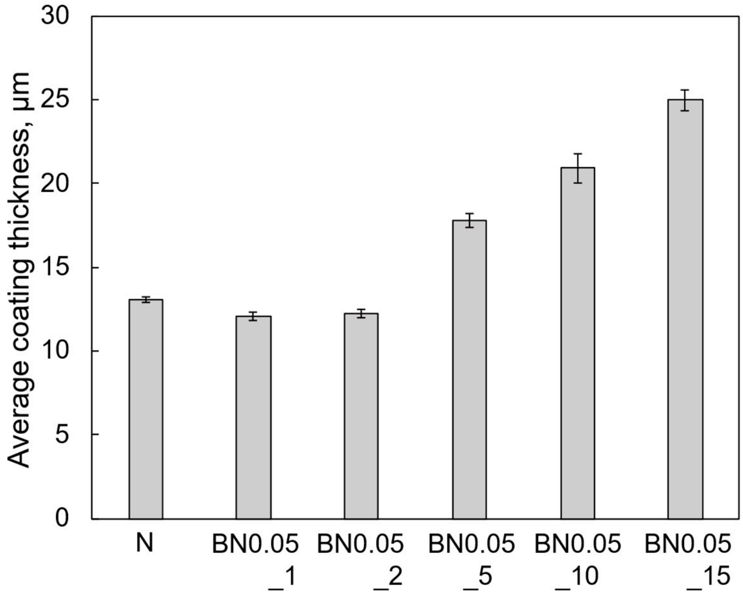

Figure S4. Average coating thickness of nano-hBN/si-PI composite coatings with different nano-hBN contents. The error bars indicate the standard deviation.

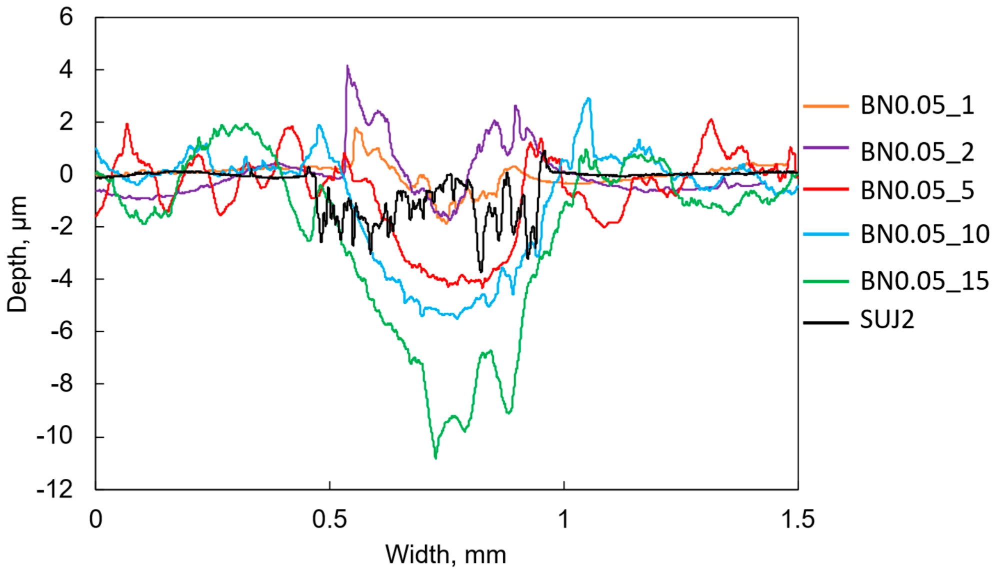

Figure S5. Cross-sectional profiles of the wear scars of the nano-hBN/si-PI composite coatings with different hBN contents and the SUJ2 substrate under unlubricated conditions.

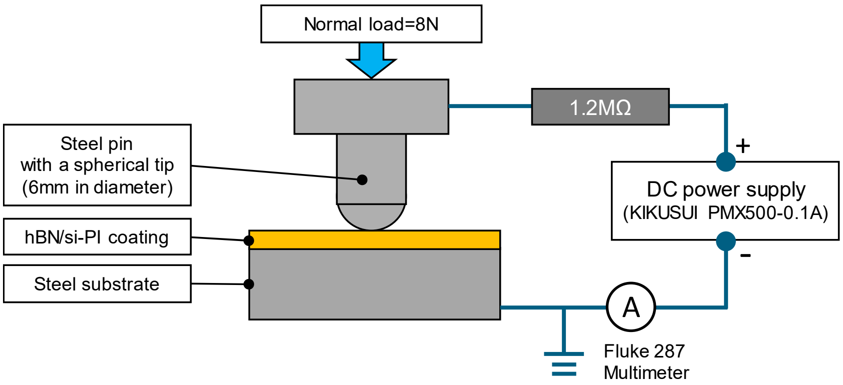

Figure S6. Measurement setup to evaluate electrical insulation of the hBN/si-PI composite coating
